# Ultra-strong tungsten refractory high-entropy alloy via stepwise controllable coherent nanoprecipitations

**DOI:** 10.1038/s41467-023-38531-4

**Published:** 2023-05-25

**Authors:** Tong Li, Tianwei Liu, Shiteng Zhao, Yan Chen, Junhua Luan, Zengbao Jiao, Robert O. Ritchie, Lanhong Dai

**Affiliations:** 1 State Key Laboratory of Nonlinear Mechanics, Institute of Mechanics, Chinese Academy of Sciences, Beijing, 100190 China; 2 School of Engineering Science, University of Chinese Academy of Sciences, Beijing, 101408 China; 3 School of Materials Science and Engineering, Beihang University, Beijing, 100191 China; 4 Department of Materials Science and Engineering, City University of Hong Kong, Hong Kong, China; 5 Department of Mechanical Engineering, Hong Kong Polytechnic University, Hong Kong, China; 6 Materials Sciences Division, Lawrence Berkeley National Laboratory, Berkeley, CA 94720 USA; 7 Department of Materials Science and Engineering, University of California, Berkeley, Berkeley, CA 94720 USA

**Keywords:** Metals and alloys, Mechanical engineering

## Abstract

High-performance refractory alloys with ultrahigh strength and ductility are in demand for a wide range of critical applications, such as plasma-facing components. However, it remains challenging to increase the strength of these alloys without seriously compromising their tensile ductility. Here, we put forward a strategy to “defeat” this trade-off in tungsten refractory high-entropy alloys by stepwise controllable coherent nanoprecipitations (SCCPs). The coherent interfaces of SCCPs facilitate the dislocation transmission and relieve the stress concentrations that can lead to premature crack initiation. As a consequence, our alloy displays an ultrahigh strength of 2.15 GPa with a tensile ductility of 15% at ambient temperature, with a high yield strength of 1.05 GPa at 800 °C. The SCCPs design concept may afford a means to develop a wide range of ultrahigh-strength metallic materials by providing a pathway for alloy design.

## Abstract (web summary)

Tungsten-based alloys with ultrahigh strength and ductility are in high demand for a wide range of applications, potentially for fusion reactors. Here the authors develop a tungsten refractory high-entropy alloy with high strength (~2.15 GPa) and sufficient ductility (~15%).

## Introduction

Materials with high performance both at room and elevated temperatures are very attractive for a wide range of critical applications, potentially for aero-engine and fusion reactors^1–4^. For decades, ultra-strong, ductile, and heat-resistant materials have long been sought by the materials community, such as tungsten-based heavy alloys and nickel-based superalloys^3,5^. However, achieving alloys with an ultrahigh strength (>2 GPa) and a sufficient ductility (>10%) has presented a long-standing challenge. This has spurred significant research into metallurgical strategies to tailor microstructures for improved properties. One classical design paradigm is utilizing dilute concentrations of alloying elements, such as Re, Ti, and Cr, to improve the properties of W-based alloys; however, the “strength-ductility trade-off” has proven to be difficult to be solved with such single principal-element alloy systems due to the problem of brittle W-enriched phases with body-centered cubic (bcc) structures^5,6^. As for nickel-base superalloys, their capacity for high-temperature resistance has been widely verified, but the stability of the high-temperature precipitate phase is still a great challenge^2,3,7^. Consequently, the development of ultra-strong and ductile heat-resisting alloys with high-temperature stability has been difficult to achieve using traditional single-principal-element metallurgical design approaches.

A emerging paradigm for the development of metallic materials has been the notion of multiple-principal-element alloys, often termed as high-entropy alloys (HEAs) or compositionally complex alloys (CCAs), which has presented promising pathways for seeking improved alloys within a previously unexplored compositional/phase space^8–13^. Numerous mechanistic phenomena have been studied to induce significant strengthening and toughening in these alloy systems, which serve to improve their strength-ductility synergy; these include high lattice friction, interstitial solid solution, grain refinement and gradients in grain size, in situ phase transformation, and nano-precipitation^14–28^. Of note here is the approach of incorporating nano-precipitates that have a positive influence on both strength and ductility. This not only results in a significant strengthening by precipitation hardening but also generates progressive multistage strain hardening that can effectively enhance ductility by delaying the onset of localized plastic instability^20,27^. However, this approach has been far less effective in refractory HEAs, which are primarily intended for high-temperature structural applications. Owing to the intrinsic brittleness of secondary phases that can form in these alloys, such as silicides, B2 and Laves phases, the majority of the previously reported refractory HEAs exhibit limited tensile ductility at ambient temperatures^13,29^. However, when the reported precipitation phase is achieved by an aging process, a single coherent precipitate or simultaneously emerging dual or hierarchical precipitations can ensure, which have achieved high enhancement effects^20,26,27^. With the single precipitation process though, the potency of the precipitation strengthening is still limited. Instigating a sequence of multiple precipitations, however, is difficult to accurately control as intermetallic compounds can readily form at grain boundaries to result in material brittleness^26–28^. Accordingly, the question that arises is whether independently controllable multiple coherent precipitation structures can be tailored by stepwise thermo-mechanical processes to generate ultrahigh strength and ductile refractory HEAs.

In this work, we utilize the immense compositional space of multiple-principal-element alloys to develop a promising tungsten-based refractory HEA with a dual-phase structure. We show that by exploiting the high melting-temperature alloy characteristics of this HEA and modulating its composition, it is possible to generate a stepwise sequence of disparate coherent nano-precipitates over different phase-transition temperature ranges to achieve a combination of ultra-high strength and tensile ductility at both ambient and elevated temperatures (800 °C). Specifically, by departing from the traditional W-Fe-Ni compositions of tungsten heavy alloys, we design a (W_1.5_Ni_2.25_Fe)_95_Ta_5_ (at.%) refractory HEA with a stepwise and independently controllable dual coherent precipitation structure. With this strategy, we first control the Ni/Fe ratio to be between 2 and 4 to avoid the formation of the topologically close-packed μ phase at phase boundaries^29,30^. Second, we add 5 at.% Ta to obtain a supersaturated solid-solution matrix for the precipitation of the coherent Ni_3_Ta-based δ phase with a D0_a_ structure and the ${{{{{{\rm{\gamma }}}}}}}^{\prime\prime}$ phase with a D0_22_ structure. Based on this composition, using a multistage thermo-mechanical processing (described in the “Methods” section), we construct a sequence of stepwise controllable coherent nanoprecipitations (SCCPs) with distinct nanoscale coherent δ and ${{{{{{\rm{\gamma }}}}}}}^{\prime\prime}$ precipitates in different phase-transition temperature ranges. Based on this synergistic nanostructure, our tungsten-based refractory HEA with the independently controllable different coherent nanoprecipitations achieves ultrahigh yield strength in excess of 2 GPa with a ~15% uniform tensile ductility at ambient temperature, and retains a high yield strength of 1.05 GPa at 800 °C.

## Results

**Fig. 1:**
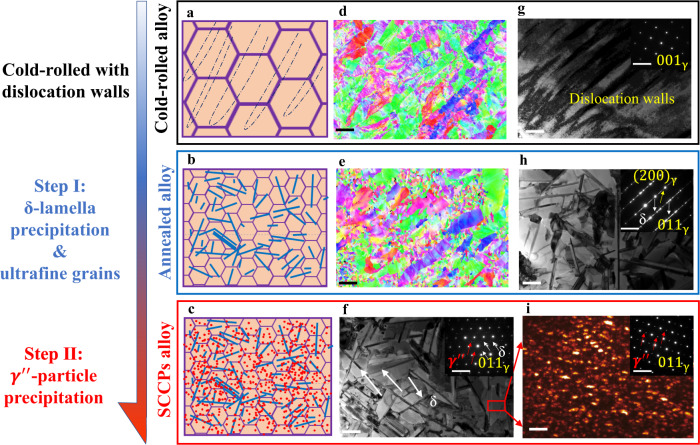
Microstructures of the cold-rolled alloy, annealed alloy, and SCCPs alloy. **a**–**c** Schematic drawings the evolution of stepwise controllable coherent nanoprecipitations. **d**, **e** EBSD maps of alloys subject to cold-rolling and annealing, respectively. **f**–**h** Bright-field (BF) TEM images and corresponding SAED patters reveal the specific structure evolution. **i** Dark-field (DF) TEM image and SAED pattern of the selected area in (**f**) show the distribution of ${{{{{{\rm{\gamma }}}}}}}^{\prime\prime}$-particles along the zone axis <011> of the fcc matrix. The scale bars in (**d**), (**e**), (**f**–**h**), and (**i**) are 10 μm, 300 nm, and 5 nm, respectively. The scale bars of SAED patterns are 10 nm^−1^.

Figure 1 shows the typical microstructural evolution of the model SCCPs alloy during stepwise thermo-mechanical processing. The initial as-cast structure of the (W_1.5_Ni_2.25_Fe)_95_Ta_5_ alloy has a dual-phase structure with bcc (W enriched) and face-centered cubic fcc (Ni-Fe-Ta enriched) phases (Supplementary Figs. 1 and 2). Schematic diagrams of the evolution of its stepwise precipitation process, from the cold-rolled material to the fully processed SCCPs alloy, are shown in Fig. 1a–c. We carefully characterize the evolution of the SCCPs structure in Fig. 1d–i, with electron backscatter diffraction (EBSD), transmission electron microscopy (TEM) and corresponding selected area electron diffraction (SAED) (details are given in the Methods section). Based on the heavy cold-rolled process, the dislocation density in the material increases sharply (Fig. 1d), forming high-density dislocation walls (Fig. 1g), which provide a structural basis for the subsequent stepwise controllable precipitation where the alloy undergoes substantial microstructure evolution during the thermo-mechanical processing, as described as follows:

Step I: δ-lamella precipitation and ultrafine grains. Coherent δ nano-lamellae appeared rapidly during the initial stage of annealing at 900 °C (Fig. 1h and Supplementary Fig. 3a). Recrystallization then occurred with the δ-lamella as the source of the nucleation and growth of the fcc grains (Fig. 1e and Supplementary Fig. 3b); the grain size eventually stabilized at below 1 μm due to a dispersion of the stable δ phase (Fig. 1e and Supplementary Fig. 3c). After annealing at 900 °C for 1 h, the alloy was fully recrystallized with an fcc grain size of ~1.5 μm (Supplementary Fig. 3d). High-angle annular dark field (HAADF) images (Supplementary Fig. 4a) show that the width of the δ layer is ~40 nm with a length ranging from 0.5 to 1 μm. Scanning transmission electron microscopy/energy-dispersive x-ray spectroscopy (STEM-EDS) maps in Supplementary Fig. 4a show that the nano-lamellar δ precipitates are rich in Ni, W and Ta yet depleted in Fe^30^. Corresponding SAED patterns (inset in Fig. 1h) revealed an ordered D0_a_ structure of this nano-lamella, as seen from the {011} zone axis of the fcc matrix.

Step II: ${{{{{{\rm{\gamma }}}}}}}^{\prime\prime}$-particle precipitation. After aging at 650°C, nano-granular ${{{{{{\rm{\gamma }}}}}}}^{\prime\prime}$ phases formed successively. The morphology and ordered D0_22_ structure of the high density ${{{{{{\rm{\gamma }}}}}}}^{\prime\prime}$ particles were revealed by dark-field (DF) TEM imaging (Fig. 1i) of the selected area in Fig. 1f, taken from the {011} superlattice reflection along the 〈011〉 zone axis of the fcc matrix. In addition, an ultrafine grain structure (Fig. 1e and Supplementary Fig. 2d, e) was observed, which remained unchanged after aging at 650°C (Supplementary Fig. 2h). EDS maps in Supplementary Fig. 4b indicate that the composition of the ordered ${{{{{{\rm{\gamma }}}}}}}^{\prime\prime}$ precipitates is close to that of the δ-lamellae, suggesting a (Ni, Fe)_3_(Ta, W)-type D0_22_ structure^29,30^. The distribution of ${{{{{{\rm{\gamma }}}}}}}^{\prime\prime}$ can be observed in the corresponding HAADF image (Supplementary Fig. 4b), where the size of these D0_22_ spheroid precipitates is ~10 nm. The precipitation sequence was also revealed by x-ray diffraction (XRD) (Supplementary Fig. 5).

**Fig. 2:**
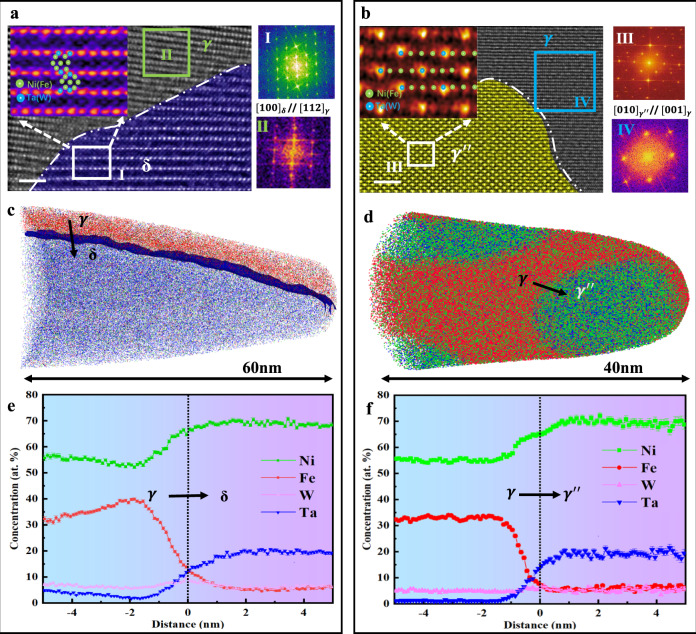
Atomic structures of the SCCPs alloy. **a**, **b** Atomic-resolution HAADF-STEM images and corresponding FFT patterns of δ, ${{{{{{\rm{\gamma }}}}}}}^{\prime\prime}$ and the fcc phase along different zone axes show the coherent relationship and specific coherent interfaces of the two different precipitations. I, II, III, and IV are the fast Fourier transforms of the corresponding regions in (**a**) and (**b**), respectively. An accurate atomic placeholder map is also provided in the correlative illustrations (red balls represent heavy elements and green ones represent light elements). **c**, **d** 3D-APT atom maps show the elemental distribution of δ-lamellae and ${{{{{{\rm{\gamma }}}}}}}^{\prime\prime}$-particles together with the 65 at.% Ni iso-concentration surfaces. **e**, **f** Proximity histograms show the elemental partitioning between the matrix and δ-lamellae and between the matrix and ${{{{{{\rm{\gamma }}}}}}}^{\prime\prime}$-particles, respectively. The scale bars in (**a**) and (**b**) are 1 and 2 nm, respectively.

The periodicity in atomic intensity was mapped by the high-resolution (HR) HAADF-STEM images, which are generated by the difference in Z-contrast among neighboring atomic columns. These results show the orientation relationships between the precipitates and matrix with the regular occupation of different atoms in the superlattice structure. Specifically, the illustration in Fig. 2a, b exhibits different highly chemically ordered precipitates with the (Ni, Fe)_3_(Ta, W)-type D0_a_ and D0_22_ structure. Fast Fourier transform (FFT) patterns of the selected area of the δ (I inset in Fig. 2a) and fcc (II inset in Fig. 2a) phases indicate that the crystal orientation relationship between the δ nano-lamellae and fcc matrix is ${[100]}_{{{{{{\rm{\delta }}}}}}}$//${[112]}_{{{{{{\rm{\gamma }}}}}}}$. In this crystallographic orientation, the δ phase has a similar atomic arrangement to the fcc matrix to generate a coherent interface^31^. The growth of δ in this crystallographic direction is limited by the small elastic distortion energy at the coherent interface, which leads to the lamellar structure^29^. The atom placeholder information of light (Ni/Fe shown as green balls) and heavy (Ta/W shown as red balls) atoms is shown in the inset in Fig. 2a. Corresponding atomic EDS maps (Supplementary Fig. 6) show that Ni/Fe atoms mainly occupy the middle position of the two rows of Ta/W atoms. With the FFT images, we characterized the crystal orientation relationship between the ${{{{{{\rm{\gamma }}}}}}}^{\prime\prime}$ phase and matrix to be ${[010]}_{{{{{{{\rm{\gamma }}}}}}}^{\prime\prime}}$// ${[001]}_{{{{{{\rm{\gamma }}}}}}}$ (Fig. 2b). The ${{{{{{\rm{\gamma }}}}}}}^{\prime\prime}$ precipitates are uniformly dispersed in the fcc matrix with almost completely coherent interfaces (Fig. 2b and Supplementary Fig. 8a). The lattice parameters of the ${{{{{\rm{\delta }}}}}}$ and ${{{{{{\rm{\gamma }}}}}}}^{\prime\prime}$ phases were directly measured from the HAADF-STEM images (Fig. 2a and b) as ${{{{{{\rm{a}}}}}}}_{{{{{{\rm{\delta }}}}}}}=\,$5.10 Å, ${{{{{{\rm{b}}}}}}}_{{{{{{\rm{\delta }}}}}}}=\,$4.24 Å, ${{{{{{\rm{c}}}}}}}_{{{{{{\rm{\delta }}}}}}}=\,$4.52 Å, ${{{{{{\rm{a}}}}}}}_{{{{{{{\rm{\gamma }}}}}}}^{\prime\prime}}=\,$3.62 Å, and ${{{{{{\rm{c}}}}}}}_{{{{{{{\rm{\gamma }}}}}}}^{\prime\prime}}=\,$7.31 Å. The axial ratio of the ${{{{{{\rm{\gamma }}}}}}}^{\prime\prime}$ unit cell is very close to 2, so its ellipsoid shape can be ascribed to the high elastic distortion energy in the [001] direction. Corresponding EDS maps (Supplementary Fig. 7) show the specific atomic placeholders. The specific atomic site occupation in the [001]$\,{{{{{{\rm{\gamma }}}}}}}^{\prime\prime}$//[001] ${{{{{\rm{fcc}}}}}}$ direction was inserted in Fig. 2b.

We next performed atom probe tomography (APT) to precisely measure the chemical composition and elemental partitioning of the two types of nano-precipitates (Fig. 2 c–f). Figure 2c, d shows representative reconstructed APT datasets of the δ-lamellae and γ“-particles, which are visualized by the 65 at.% Ni iso-concentration surfaces. The atom maps of the solute elements in the δ-lamellae and γ“-particles are displayed in Supplementary Figs. 9 and 10, respectively, which reveal that the two types of nano-precipitates are both enriched in Ni and Ta and depleted in Fe. Proximate histograms of the δ-lamellae and γ“-particles are shown in Fig. 2e, f, respectively, which illustrate the variation of solute concentrations across the interface between the precipitates and matrix. From the proximity histograms, the composition of the δ and ${{{{{{\rm{\gamma }}}}}}}^{\prime\prime}$ precipitates were both identified as (Ni_70_Fe_5_)_3_(Ta_20_W_5_), which is consistent with that the D0_a_ and D0_22_ phases. It is worthy to point out that although the δ and ${{{{{{\rm{\gamma }}}}}}}^{\prime\prime}$ precipitates have a similar composition, they have different morphologies; the former has a lamellar structure, whereas the latter has a spheroidal shape. The above results provide compelling evidence for the formation of nanoprecipitation of the coherent δ and ${{{{{{\rm{\gamma }}}}}}}^{\prime\prime}$ phases in the W-based refractory HEA, which, to our best knowledge, has not been realized before.

**Fig. 3:**
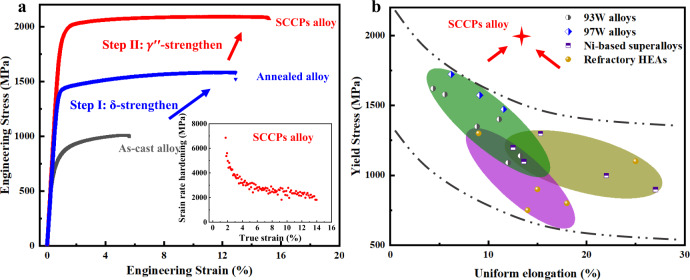
Uniaxial tensile properties of samples at different processing conditions at ambient temperature. **a** Engineering tensile stress-strain curves of the as-cast alloy, annealed alloy, and the SCCPs alloy. The inset presents the strain-hardening rate (${{{{{\rm{d}}}}}}{{{{{\rm{\sigma }}}}}}/{{{{{\rm{d}}}}}}{{{{{\rm{\varepsilon }}}}}}$) of the SCCPs alloy. **b** Maps of yield strength vs. uniform elongation of W-based heavy alloys, Ni-based superalloys, and Refractory HEAs^1–6^.

To evaluate the specific effects of the SCCPs strategy on the mechanical performance of the W-based refractory HEA, we conducted uniaxial tensile tests; the results are shown in Fig. 3a. The yield strength, σ_y_, and uniform elongation, ε_ue_, of the as-cast structures were ~600 MPa and ~7%, respectively. The tensile stress-strain curve for the annealed alloy shows a significantly higher yield strength of ∼1.4 GPa, with a tensile uniform elongation of ∼13%. The high strength of this sample is testament to the fact that the designed alloy exhibits a high hardening effect during the step I process with δ-lamella precipitation. With the subsequent ${{{{{{\rm{\gamma }}}}}}}^{\prime\prime}$-strengthen (Step II), the yield strength of the material can be further increased to 2.0 GPa with an ultimate tensile strength of 2.15 GPa without compromising the tensile ductility, which actually increases to ~15%. The strain-hardening rate as a function of strain for the samples of the SCCPs alloy, shown in the inset of Fig. 3a, indicates that this condition retains a high strain-hardening rate even at high stress levels. We also evaluated the thermal response of our W-based refractory HEA by measuring the variation in yield strength at elevated temperatures (Supplementary Fig. 11a). Notably, a yield strength above 1 GPa is maintained at temperatures as high as 800 °C due to sluggish grain growth (Supplementary Fig. 12). Indeed, the strength of our HEA favorably compares with that of other conventional superalloys and reported refractory HEAs at temperatures ranging from 25 °C to 800 °C (Supplementary Fig. 12b). We believe that the combination of yield strength, tensile strength and tensile ductility displayed by the W-based refractory HEA with the SCCPs structure is superior to previously reported tungsten-based, Ni-based superalloys, and refractory HEAs (Fig. 3b).

## Discussion

**Fig. 4:**
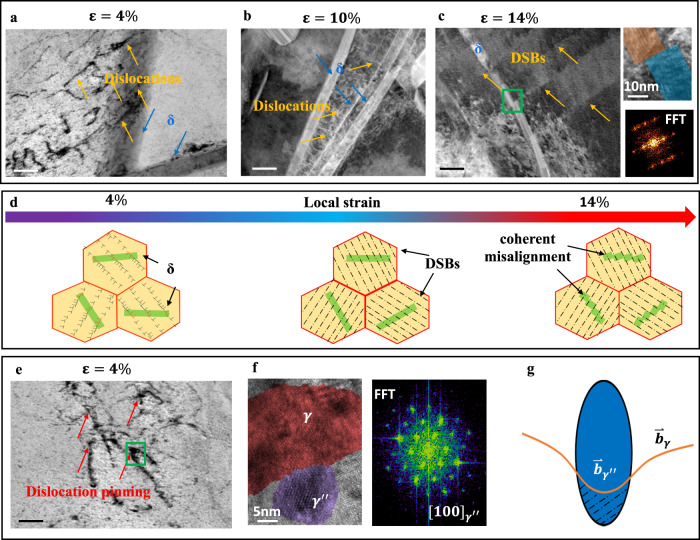
Micro-mechanisms of plastic deformation in the W-based HEA. **a**–**c** TEM and HAADF-STEM images show plastically deformed δ-lamellae at various tensile strains at room temperature. **a** Deformation microstructure at the 4% tensile strain reveals that the pronounced dislocation activity in steadily cutting through the δ-lamellae. **b** Images of dislocation slip bands (DSBs) during tensile deformation at the 10% strain. **c** Misalignments of the δ-lamellae within a grain at 14% strain and the corresponding HRTEM. **d** Schematic diagram showing the interaction of dislocations with δ-lamellae. **e** Dislocation motion is pinned by ${{{{{{\rm{\gamma }}}}}}}^{\prime\prime}$-particles at 4% strain. **f** Corresponding HRTEM and FFT images of ${{{{{{\rm{\gamma }}}}}}}^{\prime\prime}$ in (**e**). **g** Schematic diagram of the interaction between a dislocation and particle, consistent with the shearing of coherent (${\mathop{{{{{{\bf{b}}}}}}}\limits^{ \rightharpoonup }}_{{{{{{\boldsymbol{\gamma }}}}}}}\approx {\mathop{{{{{{\bf{b}}}}}}}\limits^{ \rightharpoonup }}_{{{{{{{\boldsymbol{\gamma }}}}}}}^{\prime\prime}}$) particles. Unless otherwise indicated, the scale bars are all 50 nm.

Through TEM observations, we carefully characterized the evolution of the microstructure to discern the salient strengthening mechanisms generated by the SCCPs structure. Dislocation behavior at strains of 4%, 10%, and 14% in the W-based refractory HEA with the SCCPs structure are shown in Fig. 4. Figure 4a–c, e reveal that dislocations cut through the δ-lamellae, yet are pinned by the ${{{{{{\rm{\gamma }}}}}}}^{\prime\prime}$ particles. For the coherent δ-lamellae structure, as the tensile stress increases up to the yield stress, dislocation slip occurs in the fcc matrix and involves shearing across the δ-lamellae, which indicates an early stage of the planar dislocation slip across the coherent δ/γ boundaries (Fig. 4a). With further increase in strain to 10%, slip bands can clearly be observed across the δ-lamellae (Fig. 4b). At a strain of 14%, shearing of the δ-lamellae by the slip bands leads to a large local shear (~40%) (Fig. 4c), rather than the creation of a crack, which alleviates the local stress concentration. A schematic diagram of the interaction of dislocations with δ-lamellae is shown in Fig. 4d.

For alloys with a nano-precipitation structure, the nucleation and stabilization of the nano-precipitates exert an essential effect on their mechanical properties. Hence, we carefully characterized the evolution of the SCCPs structure at different temperatures. Most importantly, the growth and coarsening of the δ and ${{{{{{\rm{\gamma }}}}}}}^{\prime\prime}$ phases, shown in Fig. 2c–f, are both controlled by the sub-micrometer diffusion, which indicates that the formation of both type of precipitates is the result of a diffusional transformation. During such processes, owing to rapid pipe diffusion, the δ-lamellae nucleated promptly at dislocation pile-ups when annealed at 900 °C (Supplementary Fig. 3a)^11,31–33^. This effect results in the homogeneous distribution of δ-lamellae within the fcc grains, instead of precipitation at grain boundaries that can lead to intergranular embrittlement in traditional alloys, such as Ni-base superalloys^31,33,34^. Remarkably, the δ phase showed excellent high-temperature stability. After annealing at 800 °C for 50 h (Supplementary Fig. 12b), both the grain size and δ phase were still stable. These are the salient factors that explain why the alloy can still maintain high strength at elevated temperatures. During the subsequent aging process, ${{{{{{\rm{\gamma }}}}}}}^{\prime\prime}$ homogeneously precipitated within the fcc grains. Indeed, we examined the stability of this phase at elevated temperatures and found that there was no substantial change in size of the ${{{{{{\rm{\gamma }}}}}}}^{\prime\prime}$ particles after aging at 800°C for 40 h (Supplementary Figs. 12b and 16b). These results indicate that both the SCCPs structure and grain size are stable at temperatures of up to 800 °C.

With the aim of deciphering the origin of the unusual mechanical behavior of the tungsten-based HEAs, we carefully investigated the precipitation hardening induced by the SCCPs structure. Because of its coherent interface, the primary strengthening feature of the δ-lamellae can be deemed to be the hinderance of dislocation motion by the δ/fcc lamellar boundaries^20,29,35^. As the parameter controlling strengthening is related to the thickness of the δ lamellae, the Hall–Petch relationship can be used to describe the strengthening effect of these dual-phase nano-lamellar structures^36^. Based on such modeling, we estimate that a high stress of ~425 MPa is required to shear the ordered δ-lamellae. With respect to the ${{{{{{\rm{\gamma }}}}}}}^{\prime\prime}$ phase, as the ${{{{{{\rm{\gamma }}}}}}}^{\prime\prime}$ particles were ~10 nm in size and maintained a coherent interface with low lattice misfit (~0.013) throughout the deformation process, strengthening by precipitate shearing was clearly the dominant mechanism, such that order hardening and lattice misfit strengthening can be considered as the main strengthening features of the γ′′ nano-precipitates^11,37–39^. The hardening from the γ′′ can be measured by uniaxial tensile testing to be ~600 MPa (Fig. 3a). It should be also noted that an important benefit of the coherent interfaces of both the δ-lamellae and γ′′ particles is that they do not contribute to a marked decrease in ductility of our SCCPs structure. Hence, the synergistic strengthening of the two types of nanoscale precipitates in the SCCPs structure provides a prime contribution to the overall ~1 GPa strength of the W-based refractory HEA, as quantitatively described in the “Methods” section.

Owing to the SCCP process, our W-based refractory HEA alloy not only exhibits high strength but it retains its tensile ductility, which we measured as a uniform elongation of 15%. A major reason for this is the homogeneous distribution of the SCCPs structure, which facilitates the steady accommodation of a large number of dislocations at the coherent δ/matrix interfaces during plastic deformation (Fig. 4b and Supplementary Fig. 13). In addition, because of the small size of the ${{{{{{\rm{\gamma }}}}}}}^{\prime\prime}$ particles and their similar lattice parameter to the fcc matrix, the associated elastic interaction between the ${{{{{{\rm{\gamma }}}}}}}^{\prime\prime}$ precipitates and cutting dislocations is correspondingly lowered^14,31,32,37^, thereby preventing any crack initiation at the precipitate–matrix interface during the deformation process. Moreover, the coherent misalignment of δ-lamellae (Fig. 4c) plays an important role in reducing the tendency for cleavage. When the strain exceeds 10%, the shearing of δ-lamellae by slip bands leads to coherent misalignments instead of microcrack formation. Unlike traditional W-based heavy alloys where intermetallic compounds invariably act as the origin of microcracks from major dislocation pile-ups during deformation, the coherent interface of the δ phase in the W-based HEA effectively alleviates this problem^14,39–43^. Accordingly, all these beneficial factors act in concert to contribute to the unusual combination of high strength and good ductility in our W-based HEA.

In summary, by incorporating the stepwise and independently controllable coherent nanoprecipitation strategy, a tungsten-based refractory high-entropy alloy has been successfully developed to achieve an ultrahigh strength without compromise in tensile ductility. Through carefully designed thermo-mechanical processing, the SCCPs structure has been developed to induce coherent particle hardening from the nano-precipitation of both δ-lamella and ${{{{{{\rm{\gamma }}}}}}}^{\prime\prime}$ particles within a bcc and fcc dual-phase matrix. This alloy exhibits a high tensile strength of 2.15 GPa and a tensile ductility of 15% at ambient temperatures, yet retains a tensile strength of above 1 GPa at temperatures of as high as 800 °C. We believe that our SCCPs design philosophy may provide a highly effective approach for the future development of other advanced materials with superior mechanical properties.

## Methods

### Materials

Bulk (W_1.5_Ni_2.25_Fe)_95_Ta_5_ (W30-Ni45-Fe20-Ta5, in at.%) alloy ingots were prepared by arc melting pure metals (all with purity >99.99%) in an argon atmosphere. Alloy samples were repeatedly melted at least 10 times to ensure uniform melting and improve their chemical homogeneity. After cooling, the bulk ingots with dimensions of 50 mm × 50 mm × 10 mm were homogenized at 1200 °C for 5 h in an Ar atmosphere and subsequently hot-rolled at 1200 °C with a rolling reduction ratio of 80%, i.e., with a thickness reduction from 10 to 2 mm. After hot rolling, the ingots were rolled at ambient temperature (25 °C) with a reduction ratio of 75% to reduce the thickness from 2 to 0.5 mm (this is termed the cold-rolled alloy). Following cold rolling, stepwise thermodynamic treatments involving annealing at 900 °C for 5 min (annealed alloy), then aging at 650 °C for 20 h, followed by water quenching, were carried out to obtain the SCCPs structure with δ-lamellas and ${{{{{{\rm{\gamma }}}}}}}^{\prime\prime}$ particles.

### Microstructure characterization

Electron back-scattered diffraction (EBSD) measurements were carried out in a field-emission SEM (JEOL–JSM–7001 F) equipped with an automatic orientation acquisition system (Oxford Instruments-HKL Channel 5). Specimens were mechanically ground and polished, and then electropolished at room temperature with an electrolyte of 90% ethanol and 10% perchloric acid. Constituent phases in the alloy were identified in an X-ray diffractometer (XRD; Rigaku Dmax 2500) using a CuKα radiation. The microstructures before and after deformation were characterized by scanning electron microscopy (SEM; JSM-7100F) and transmission electron microscopy (TEM; JEM-2100F) at 200 kV. HAADF images were recorded in a Cs-corrected TEM (Titan Cubed G2 60, 300 kV). The samples for TEM and HAADF were prepared by focused ion beam (FIB; FEI Helios Nanolab 600i). Chemical compositions were measured by atom probe tomography in a CAMEACA LEAP 5000XR APT. Needle-shaped APT samples were also fabricated by FIB. These APT samples were examined at 50 K in voltage mode. The pulse repetition rate was 200 kHz and the pulse fraction was 20%, with an evaporation detection rate of 0.2% atom per pulse. Three-dimensional (3D) reconstructions of the APT data were performed using Imago Visualization and Analysis Software (IVAS, version 3.8.2).

### Mechanical property evaluation

Dog-bone-shaped specimens for uniaxial tensile testing, with a gauge length of 12.5 mm and a cross-section area of 2.0 × 0.5 mm^2^, were fabricated along the longitudinal direction of the cold-rolled strip material by electro-discharge machining. Using an MTS-810 servo-hydraulic universal testing machine (MTS Corp., Eden Prairie, MN), uniaxial tensile tests were carried out at room temperature at a constant strain rate of 5 × 10^−4^ s^−1^. High-temperature tensile tests were conducted on the same MTS-810 testing machine at a strain rate of 10^−3^ s^−1^. The temperature was monitored using three K-type thermocouples. At least three samples were tested in each condition to ensure reproducibility of data.

## Supplementary information

Supplementary Information

Peer Review File

## Data Availability

The data that support the findings of this study are available from the corresponding authors on request.
