# Supplementary material for: Ultra-strong tungsten refractory high-entropy alloy via stepwise controllable coherent nanoprecipitations
Source: Nat Commun. 2023 May 25;14:3006. doi: 10.1038/s41467-023-38531-4 (PMC10213035; doi:10.1038/s41467-023-38531-4)
Supplement: Supplementary file 1 — Supplementary Information [file 41467_2023_38531_MOESM1_ESM.pdf]

# Supplementary information

# Ultra-strong tungsten refractory high-entropy alloy via stepwise controllable coherent nanoprecipitations

Tong Li, Tianwei Liu, Shiteng Zhao, Yan Chen, Junhua Luan, Zengbao Jiao, Robert O. Ritchie, Lanhong Dai.

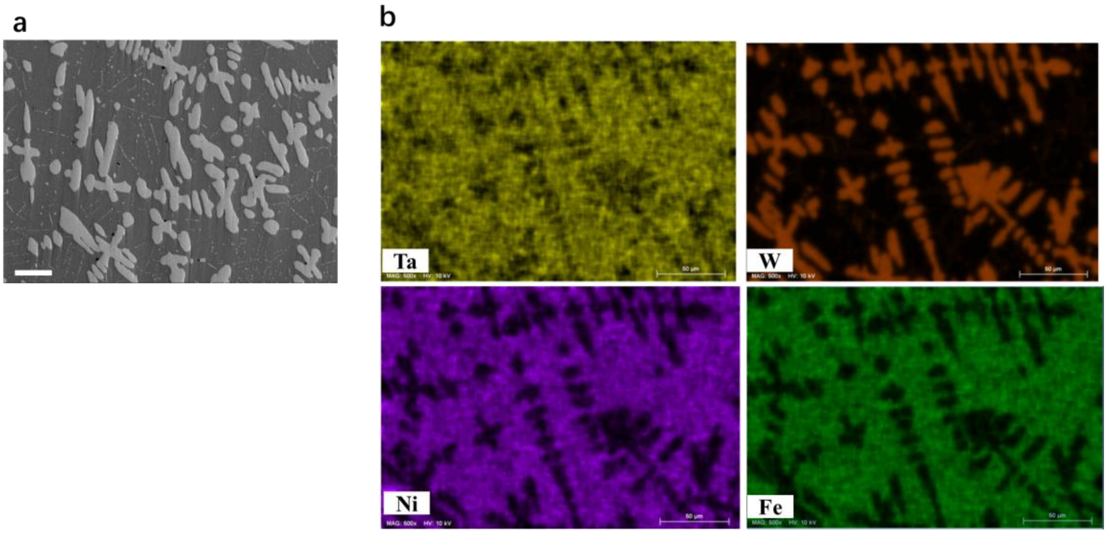


**Supplementary Fig. 1. SEM image and corresponding EDS maps. a** SEM image of base alloy in the as-cast condition. **b** Corresponding EDS maps show the distribution of Ta, W, Ni, and Fe, respectively. The scale bar is 20 μm.

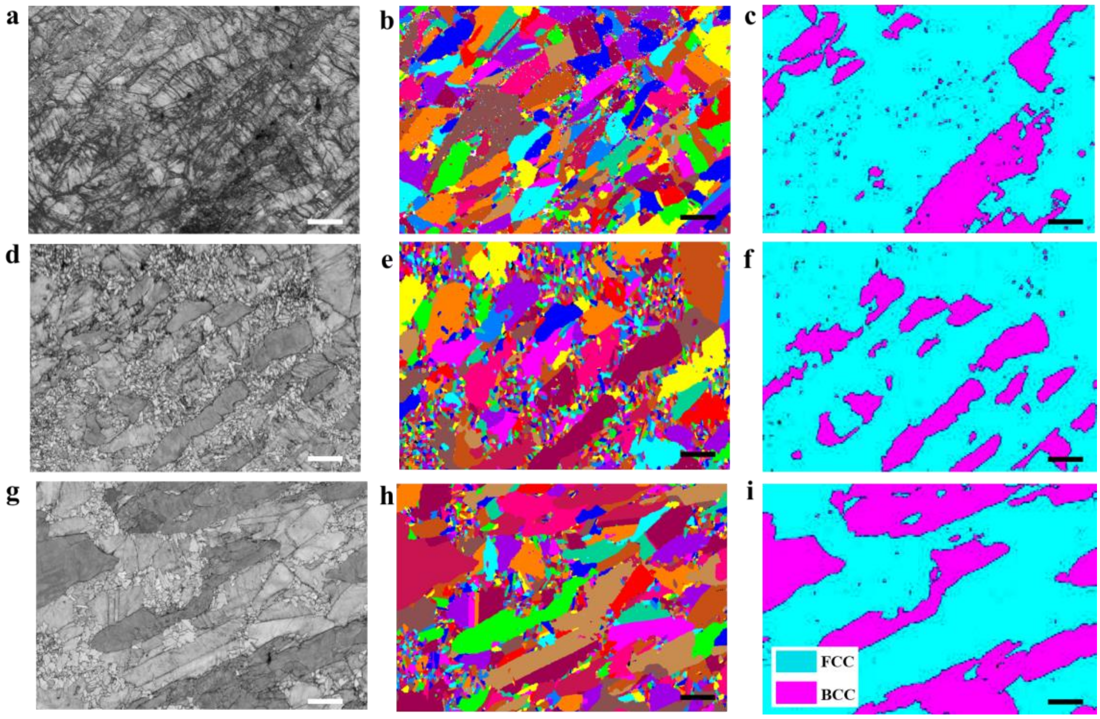


**Supplementary Fig. 2. a, d, and g are the corresponding Image Quality (IQ) patterns of Fig. 1a-c, respectively**. **b, e,** and **h** show the grain distribution of **Fig. 1a-c**. **c, f,** and **i** show the phase distribution of **Fig. 1a-c**. The scale bar is 10 μm.

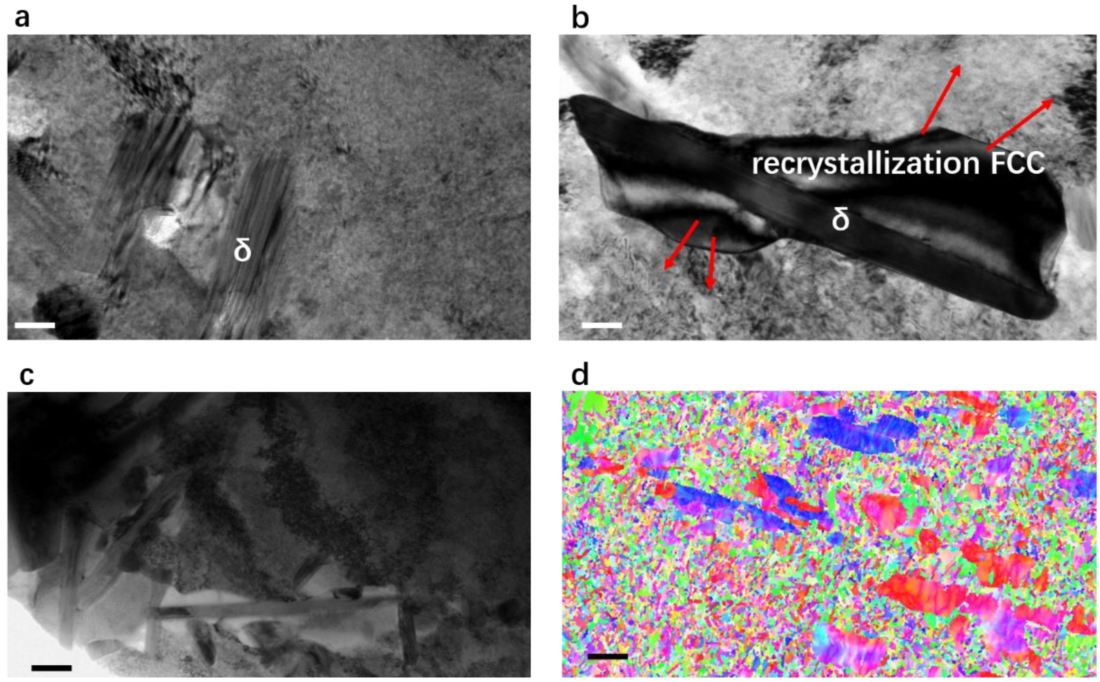


**Supplementary Fig. 3. Microstructure evolution during annealing at 900℃. a-c** TEM images correspond to increasing annealing times of 30 s, 1 min and 2 min, respectively. **d** EBSD map of the microstructure after annealing for 1 h showing the perfect recrystallization structure. The scale bars in **a-b, c**, and **d** are 50 nm, 100 nm, and 10 μm, respectively.

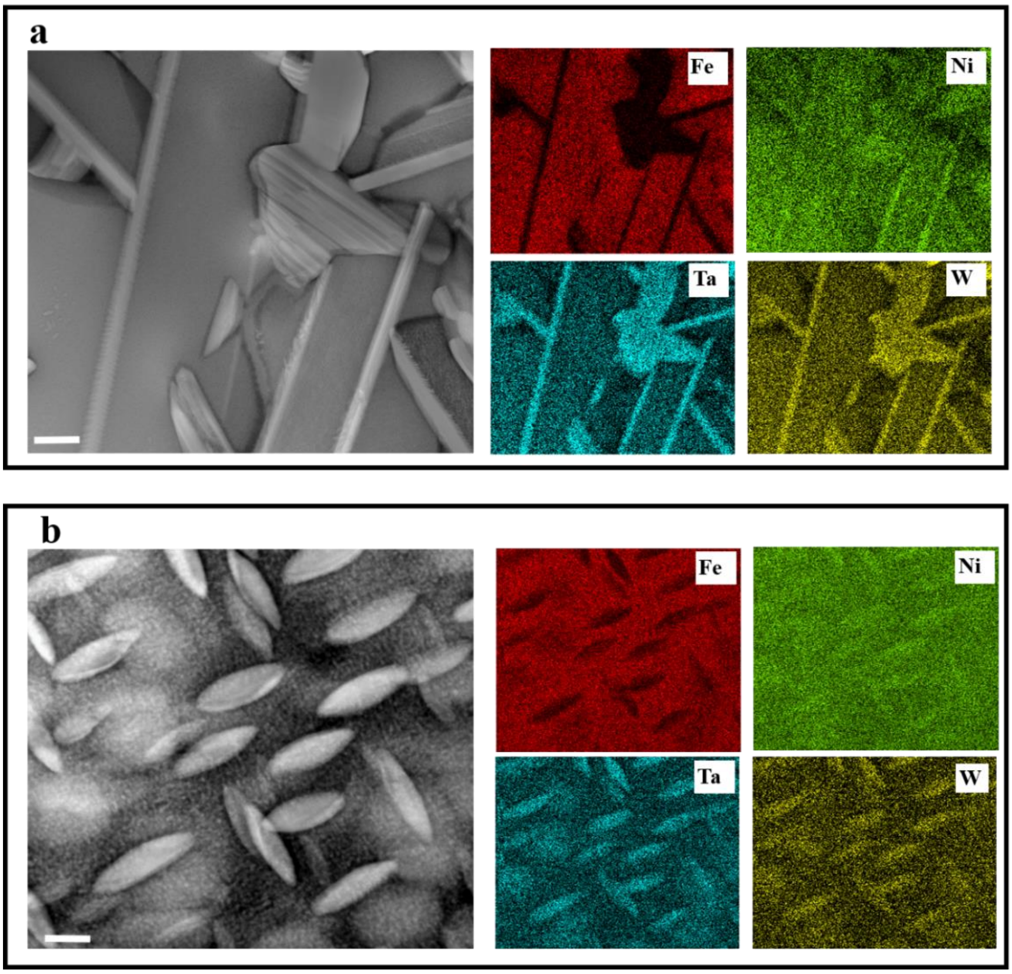


**Supplementary Fig. 4. HAADF-STEM images and corresponding EDS maps of δ-lamellae and $\gamma''$-particles.** The scale bar **a** and **b** are 100 nm and 5 nm, respectively.

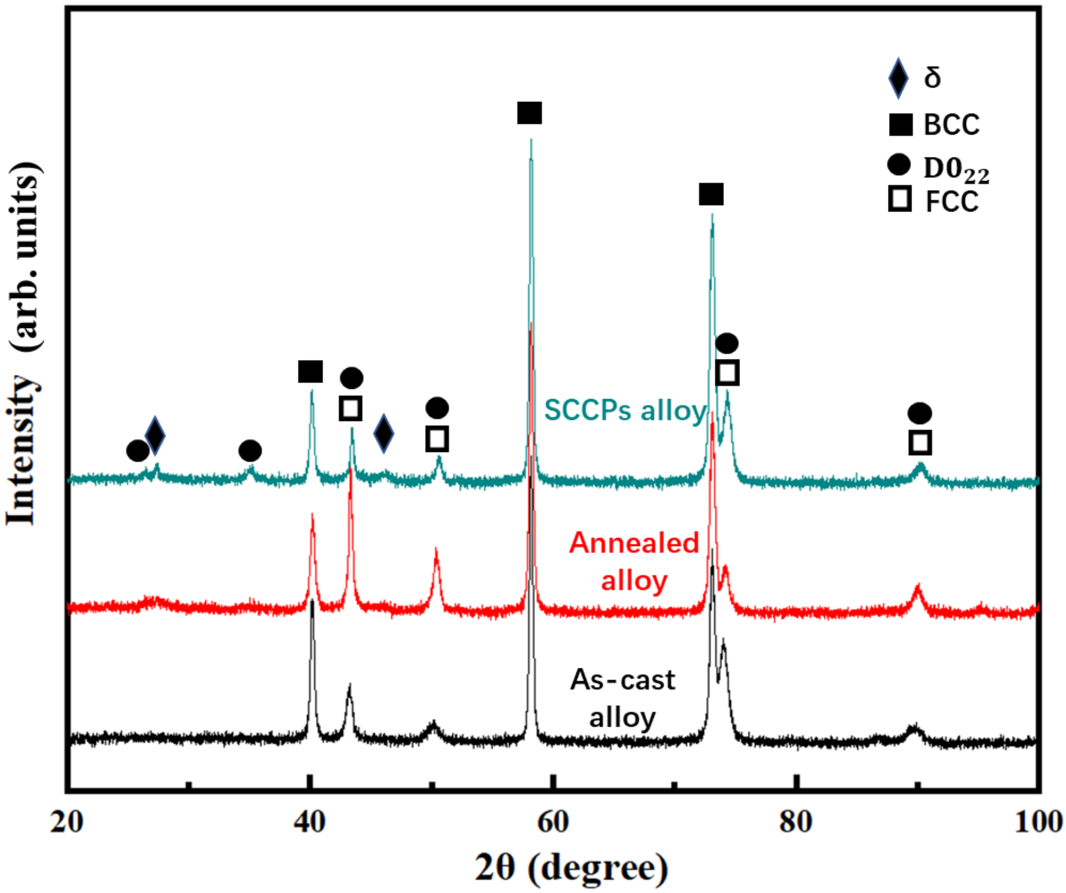


**Supplementary Fig. 5. XRD pattern of alloys at different processing states,** showing the evolution of the SCCPs in the alloy.

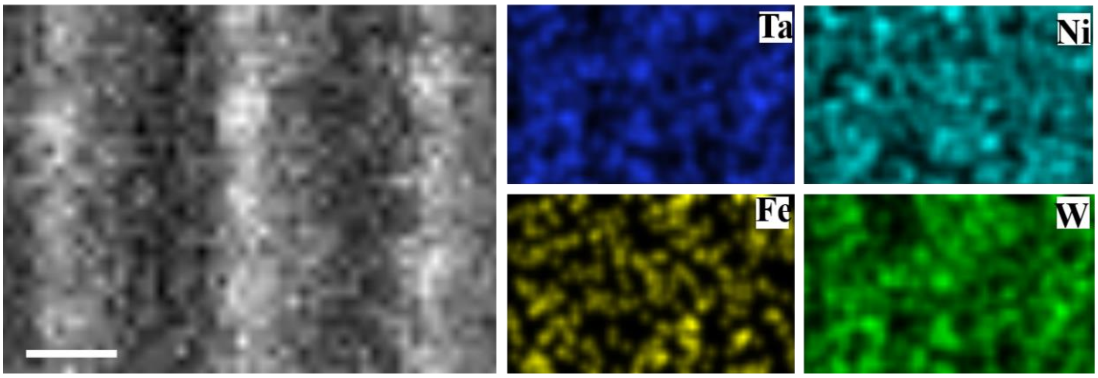


**Supplementary Fig. 6. Atomic-resolution EDS maps** corresponding to areas outlined by purple and red dashed squares in Fig. 2a. The scale bar is 50 pm.

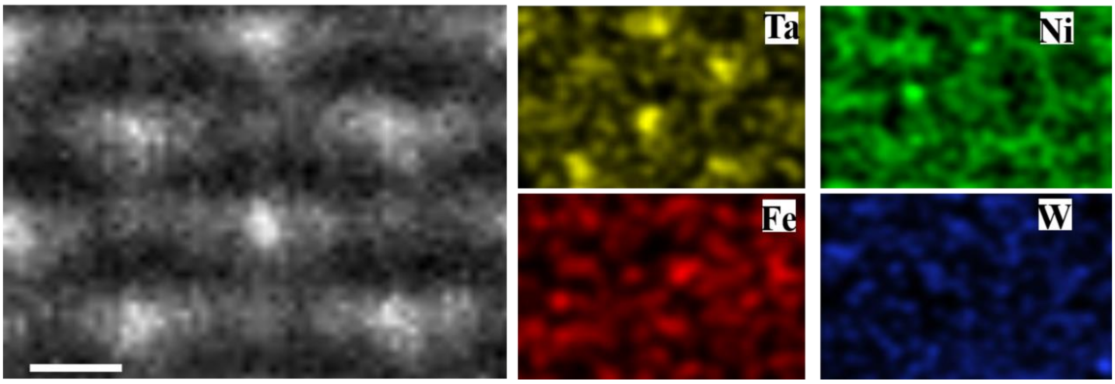


**Supplementary Fig. 7. Atomic-resolution EDS maps** corresponding to areas outlined by purple and red dashed squares in Fig. 2b. The scale bar is 50 pm.

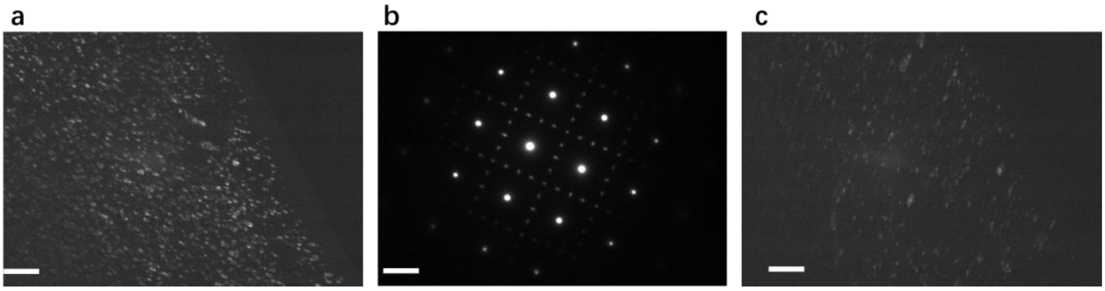


**Supplementary Fig. 8. Distribution of $\gamma''$-particles in the *fcc* matrix**. **a,c** are the dark- field TEM images of **b** along adjacent diffraction spot of $D0_{22}$. The scale bars of dark- field TEM images and SAED pattern are 50 nm and 5 $nm^{-1}$, respectively.

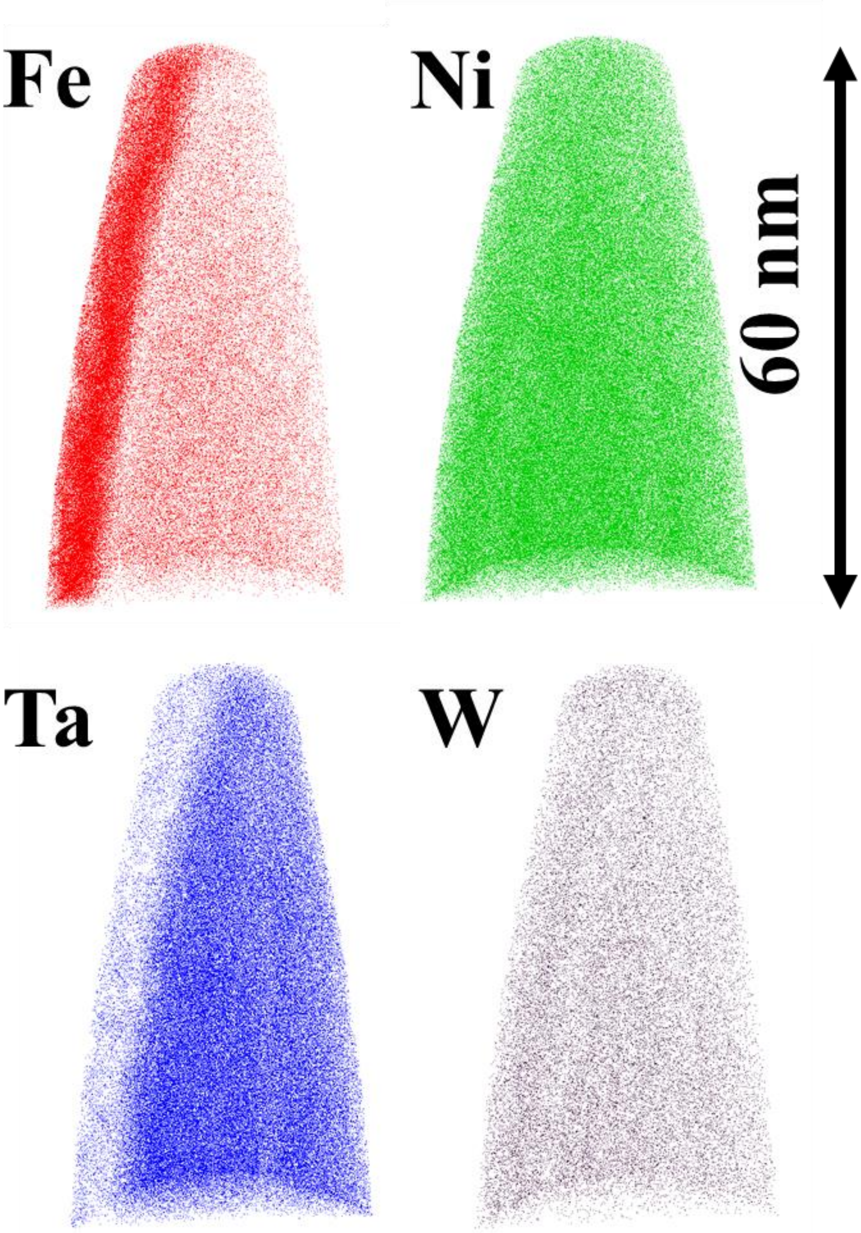


**Supplementary Fig. 9. Atom probe tomography images** showing the distribution of each element in the δ-lamellae shown in Fig. 2c.

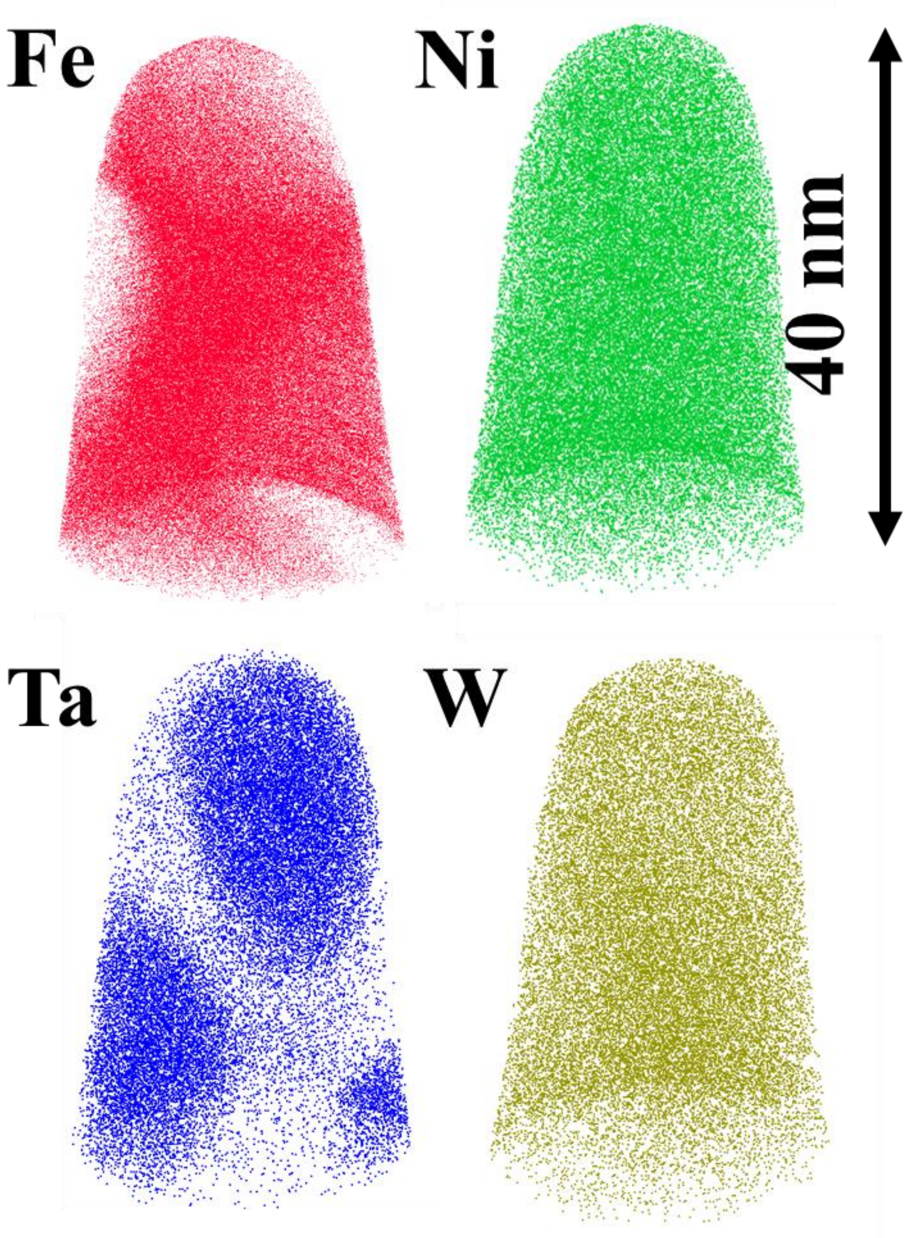


**Supplementary Fig. 10. Atom probe tomography images showing the distribution of each element in the $\gamma''$-particles shown in Fig. 2d.** The precipitates can be seen to be Ni and Ta rich but correspondingly depleted in Fe.

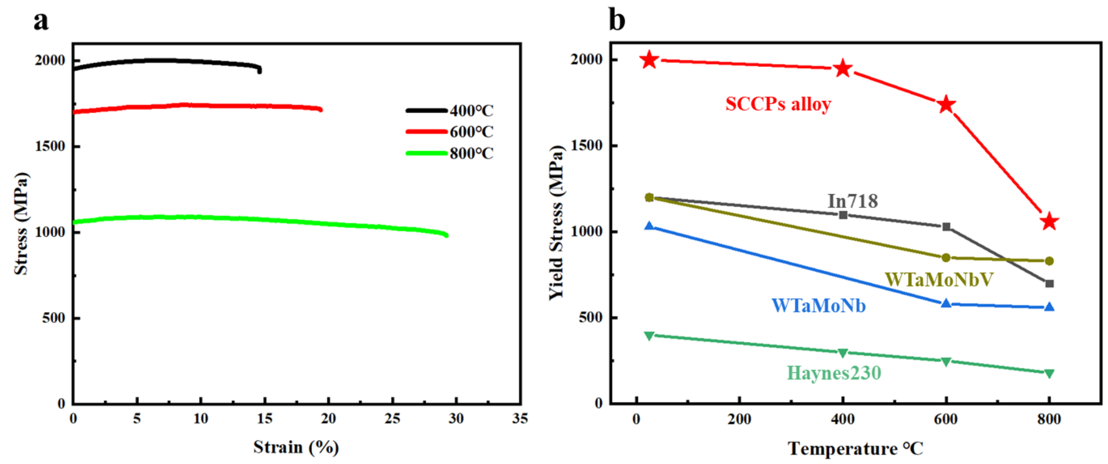


**Supplementary Fig. 11. Mechanical properties of the W-based HEA at elevated temperatures. a** Uniaxial tensile engineering stress-strain curves at 400°C, 600°C, and 800°C. **b** Yield strength of the W-based HEA as a function of temperature, as compared with those of some representative high-temperature alloys[19-21].

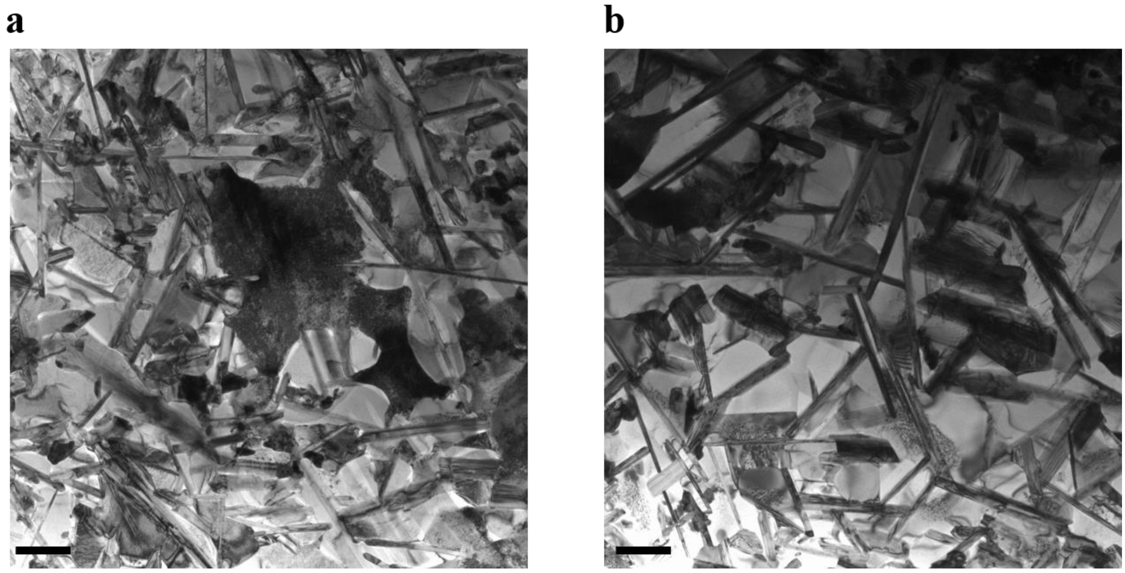


**Supplementary Fig. 12. Variation in grain size at elevated temperatures during long-duration annealing.** TEM image of the W-based HEA after annealing for 50 h at **a** 600℃, and **b** 800℃. The scale bars are 500 nm.

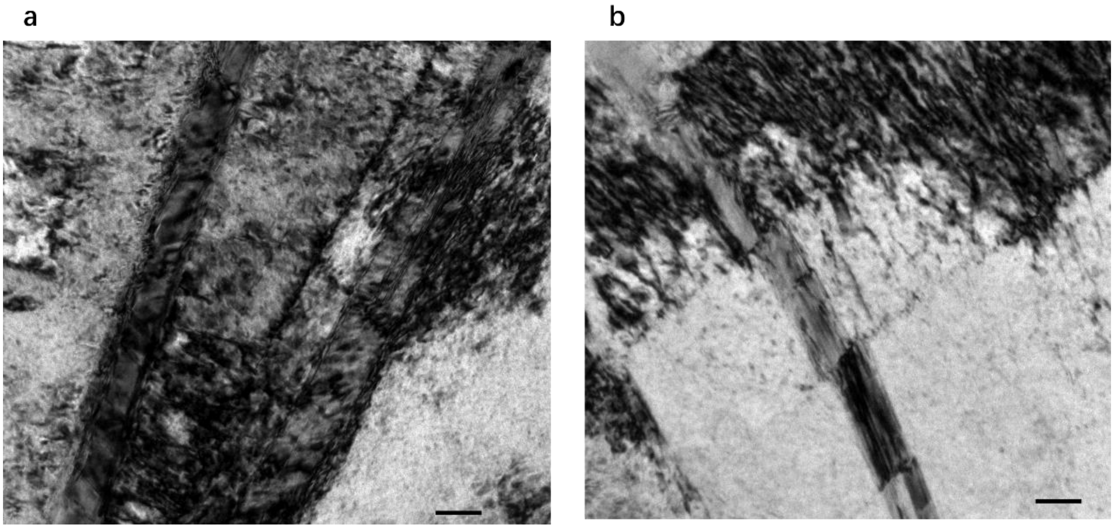


**Supplementary Fig. 13. Dislocation motion in the SCCPs alloy at 10% and 14% plastic strain.** **a** TEM image of **Fig. 4b** showing dislocations cutting through the δ-lamellae before piling-up at phase boundaries. **b** Corresponding TEM image of **Fig. 4c.** The scale bar is 50 nm.

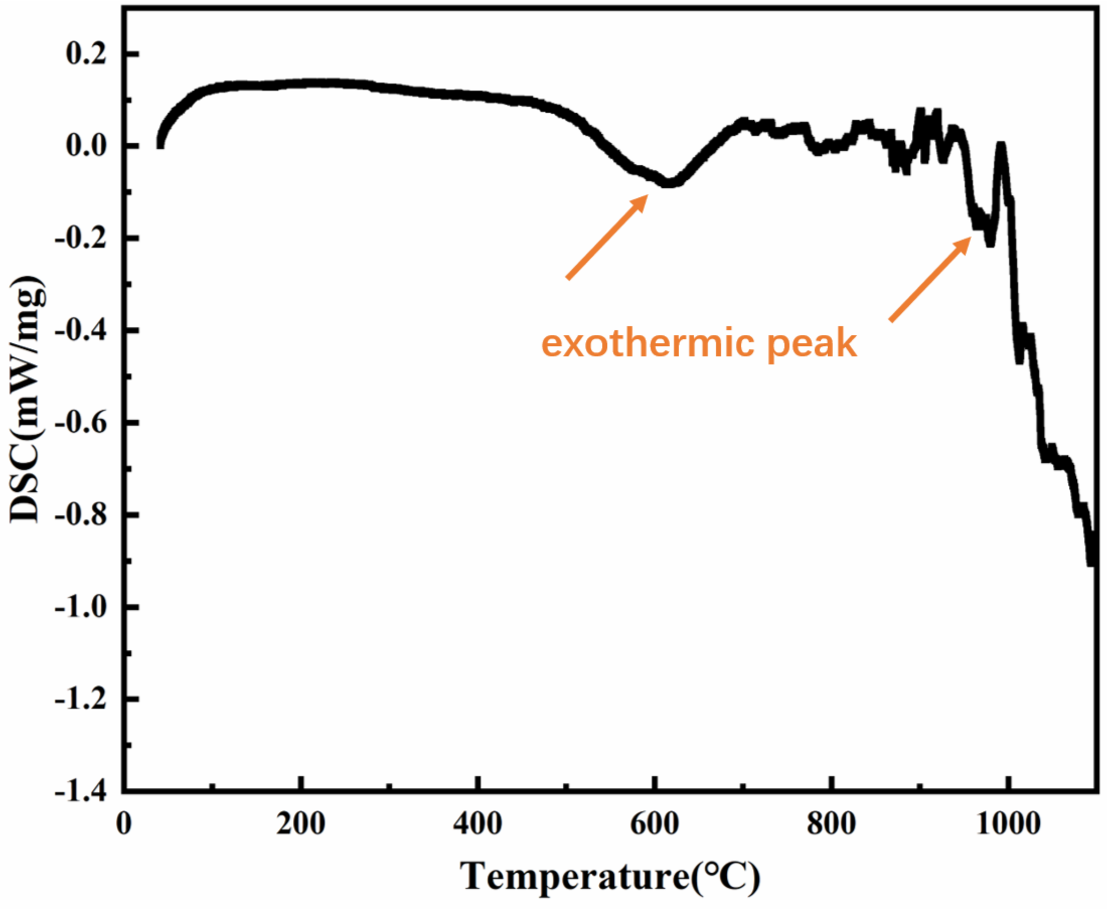


**Supplementary Fig. 14. The DSC pattern reveals the different temperature ranges of the four stepwise precipitations.**

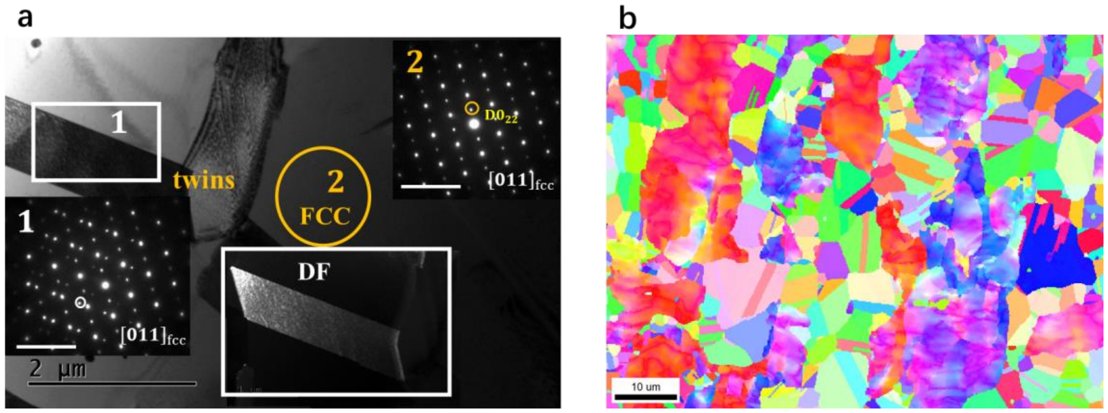


**Supplementary Fig. 15. Microstructure of the cold-rolled W-based HEA after annealing at 1200°C for 1 min. a** TEM image and SAED patterns show the *fcc* matrix structure without δ-lamella precipitation. Inset shows a dark-field image insert indicating that the $\gamma''$ particles remain uniformly distributed in the *fcc* matrix. **b** EBSD image shows the complete recrystallization occurring at high temperatures. The scale bars of SAED patterns are 10 nm$^{-1}$.

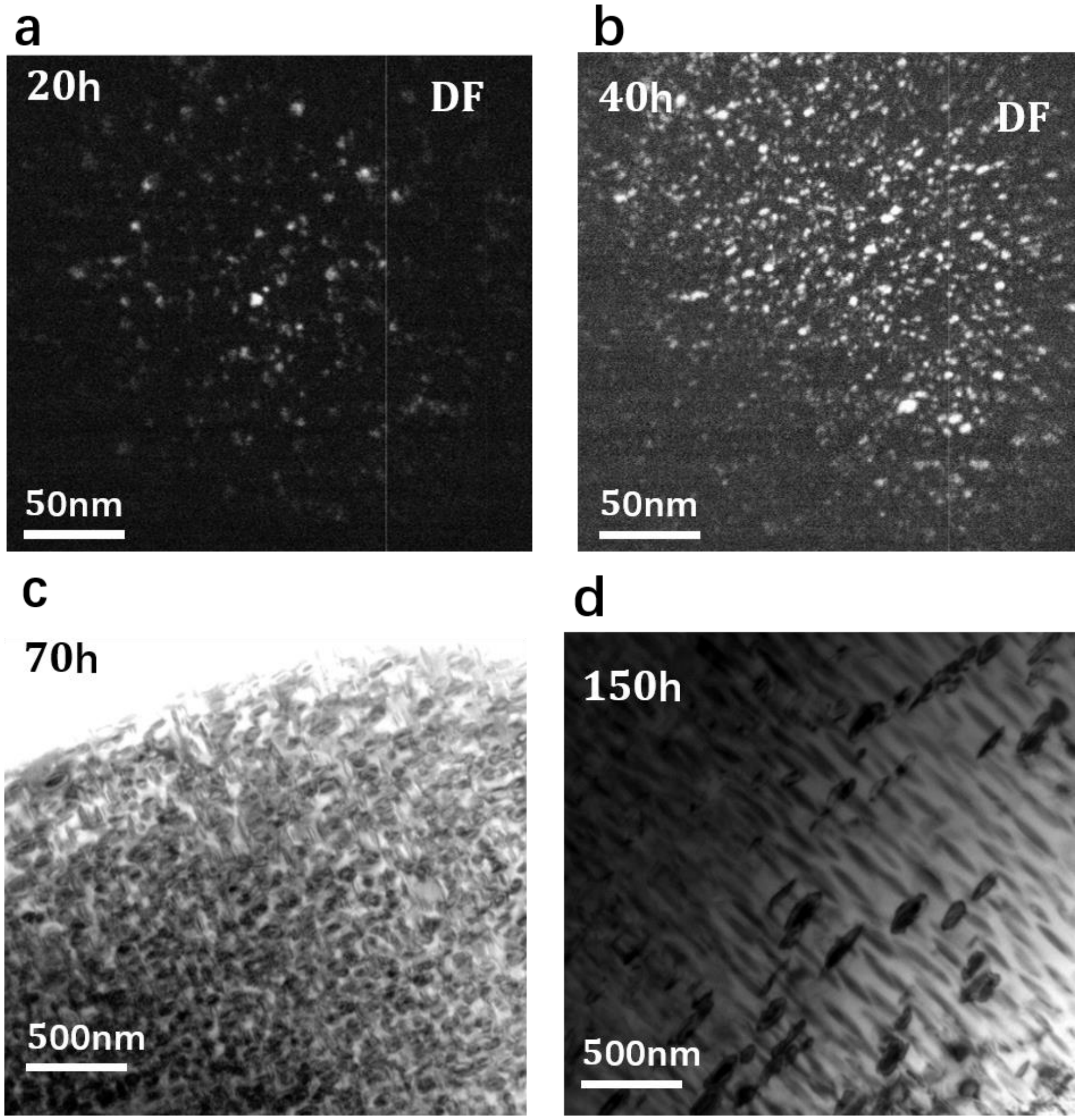


**Supplementary Fig. 16. Evolution of $\gamma'$-particles during prolonged aging. a-d** show the change in the morphology of the $\gamma'$-particles after aging for 20, 40, 70, and 150 h, respectively. The size of the precipitates is significantly larger for aging times over 40 h.

**Supplementary Table 1. Concentrations of elements in the two-phase structure after annealing at 1200°C for 5 h**

| Phase | W (at.%) | Ta (at.%) | Fe (at.%) | Ni (at.%) |
|---|---|---|---|---|
| *fcc* | 6.0 | 7.3 | 39.3 | 47.4 |
| *bcc* | 85.9 | 4.9 | 4.5 | 4.8 |

**Supplementary Note 1. Identification of matrix and precipitate phases**

All pure elements were melted together by arc melting and then cooled in an Ar atmosphere. During cooling from the liquid temperature to the liquid–solid transition temperature, phase separation took place in the liquid phase ($L_m$)[1]. During the typical dendritic solidification process, $L_m$ then decomposed into a W-rich dendritic phase with a body-centered cubic (bcc) structure and inter dendritic liquid formed face-centered cubic (fcc) phase with high concentrations of Ni and Fe and Ta. The phase transformation can be expressed as:

$$L_m \rightarrow fcc + bcc \ . \quad (1)$$

The dendritic structure of the *bcc* phase was the first to solidify during the phase transformation of $L_m$ to the solid phase. During the liquid-to-solid phase transformation, the separation of elements was very important. From the EDS data in Supplementary Table 1, the total content of W (at. %) reached in the bcc phase was approximately 85 at.%, with small amounts of Fe, Ni. The principal components of the fcc phase were Ni, Fe, and Ta, consistent with the EDS results (Supplementary Fig. 1 and Supplementary Table 1). However, small amounts (~7 at. %) of Ta and W remained in the fcc phase.

To obtain the SCCPs structure with coherent δ-lamellas and $\gamma''$ particles, the cold-rolled samples were heat-treated at different temperatures[2-7]. Differential scanning calorimetry (DSC) results in Supplementary Fig. 14 revealed a clear endothermic peak near 900° and 650°C, indicating that different solid–solid phase transformations had taken place. We concluded that the phase transitions involved the precipitation of the $Ni_3Ta$-based D0a structure (δ) and $D0_{22}$ structure ($\gamma''$)[1,5,7]. When the cold-rolled samples were annealed at 900°C for 5 min, δ-lamellae precipitated from the base fcc phase and formed the new fcc phase (*fcc1*). Subsequently, the annealed samples were aged at 650°C for 20 h during which $\gamma''$ particles precipitated from the base *fcc1* phase and formed a new fcc phase (*fcc2*). This stepwise phase transformations can be expressed as:

$$fcc \rightarrow fcc1 + \delta \ , \quad (2)$$

$$fcc1 \rightarrow fcc2 + \gamma''. \quad (3)$$

During the stepwise phase transformations, two types ordered chemical transformations occurred with coherent δ-lamellas and $\gamma''$ particles in the fcc matrix. To further analyze the stability of the δ-lamellas and $\gamma''$ particles, the materials were subjected to heat treatment at different times and temperatures. The results show that the δ-lamellae disappear with the samples undergoing complete recrystallization after annealing at 1200℃ for 1 min (Supplementary Fig. 15); this signifies that the ordering elements are re-dissolved into the matrix with increase in temperature. Because of this, the morphology and structure of δ-lamella can be controlled. Moreover, almost no growth of $\gamma''$ particles was observed when the aging time exceeded 40 h (Supplementary Fig. 16), although the $D0_{22}$ structure was maintained. Thus, it is very important to control the morphology and distribution of particle precipitation by controlling the aging time. Based on this, we can conclude that the $(W_{1.5}Ni_{2.25}Fe)_{95}Ta_5$ alloy develops its independently controllable different nanoprecipitations structure during the solidification process through one liquid–solid phase transformation and two solid–solid phase transformations.

**Supplementary Note 2. Stepwise controllable nanoprecipitations strengthening mechanisms**

There are also additional strengthening mechanisms at play in the W-based HEA alloy with our SCCPs structure which involve solid-solution hardening from the fcc matrix and Ta/W enriched bcc phase. For this, we can estimate the yield strength $\sigma_{0.2}$ from the following expression:

$$\sigma_{0.2} = \sigma_{\gamma} + \Delta\sigma_{\delta} + \Delta\sigma_{\gamma''} + \Delta\sigma_{B} \ , \quad (4)$$

where $\sigma_{\gamma}$ is the strength of the fcc matrix, $\Delta\sigma_{\delta}$ and $\Delta\sigma_{\gamma''}$ are the strengthening respectively from the coherent δ-lamellae and $\gamma''$ particles, and $\Delta\sigma_{B}$ is related to the bcc phase strengthening. The strengthening capacity provided by each phase is closely related to its volume fraction in dual-phase alloys.

The volume fractions of the fcc and bcc phases can be estimated from the SEM images (Supplementary Fig. 1) to be respectively ~65% and 35%. Thus, $\sigma_{\gamma}$ was deduced to be ~550 MPa for the Ni–Fe-based solid solution with a consistent ultra-fine grain structure[4,8]. TEM images after deformation show dislocation pile-ups at the phase boundaries between the fcc matrix and the bcc phase, an observation that suggests that the hardening from the bcc occurs by a second-phase strengthening mechanism. The contribution from the W-Ta-based bcc phase, $\Delta\sigma_{B}$, can be estimated from tungsten-based alloys to be ~350 MPa[7,9].

The specific contributions of independently controllable different nanoprecipitations with δ-lamellae and $\gamma''$ particles to strengthening the alloy are evaluated below. The strengthening by the δ-lamellae is assessed in terms of a dual-phase nano-lamellar material in which the yield strength is described by the Hall–Petch relationship[10-15]. In such a structure, dislocations initially start to propagate inside the soft phase and pile-up at interphase boundaries. Yielding occurs when the leading dislocation in a pile-up can overcome the barrier strength and transmit slip across the boundary. Therefore, the thickness and physical properties of the lamellae structure are the factors controlling this strengthening[15,16]. For the δ-lamellae precipitates, the

strengthening contributed by the δ/fcc boundaries can be evaluated by $\Delta\sigma_{\delta} = fk_{\delta}(\lambda)^{-0.5}$, where $k_{\delta}$ is the Hall–Petch coefficient, $\lambda$ is the average thickness of the δ lamellae, and $f$ is the volume fraction of δ-lamellas (estimated to be ~15% from the TEM images in Fig. 1e and Supplementary Fig. 4a). The Hall–Petch coefficient $k_{\delta}$ can be calculated as[6,17]:

$$k_{\delta} = \left(\frac{n^2 G_{\delta}{}^2 b_{\delta}{}^2}{8\lambda}\right)^{\frac{1}{2}} , \tag{5}$$

where $n$ ~ 4 is the number of dislocations crossing the same lamellae (Fig. 4a), $G_{\delta}$ is the shear modulus of the δ phase (= 172 GPa), $b_{\delta}$ is the Burgers vector of the δ phase = $\sqrt{2}a_{\delta}/2$ ≈ 0.361 nm, $a$ is the lattice constant of the δ phase (= 0.51 nm) obtained from HAADF-STEM images (Fig. 2a), and $\lambda$ is the average lamellar thickness of the δ-lamellas (~40 nm). Based on the above calculations, the strengthening contributed by the δ-lamellas can be estimated to be ~425 MPa.

To analyze the strengthening effect of the coherent $\gamma''$-particles, we consider the creation of an anti-phase boundary (APB) when dislocations shear through ordered particles on their slip planes; this is a primary factor in order strengthening[14,15], although due to the lattice misfit between the precipitates and the fcc matrix, coherency strengthening also plays an important role. Accordingly, we model to contribution from $\gamma''$-particle strengthening by considering two terms, namely from the APB ($\Delta\sigma_{pA}$) and from lattice misfit ($\Delta\sigma_{pC}$). The increment in yield strength due to strengthening from the APB, $\Delta\sigma_{pA}$, can be expressed as[15]:

$$\Delta\sigma_{pA} = M\frac{\gamma''_{APB}}{2b_{\gamma''}}\left\{\left(\frac{4\gamma''_{APB}f}{\pi T}\left(\frac{\sqrt{6}Rh}{3}\right)^{\frac{1}{2}}\right)^{1/2} - \beta f\right\} , \tag{6}$$

with the corresponding strength increment due to lattice misfit, $\Delta\sigma_{pC}$, is given by:

$$\Delta\sigma_{pC} = 1.7MG|\varepsilon|^{\frac{3}{2}}\left(\frac{h^2 f(1-\beta)}{2b_{\gamma''}R}\right)^{\frac{1}{2}} . \tag{7}$$

Here, $M$ = 3.06 is the Taylor factor, $G$ = 77 GPa is the shear modulus of the $\gamma''$, $b_{\gamma''}$=$\sqrt{2}a_{\gamma''}/2$ = is the Burgers vector of the $\gamma''$, $\gamma''_{APB}$ = 0.296 J/m$^2$ is the APB energy, which is obtained from data for $Ni_3Ta$-type $D0_{22}$ precipitates in Ni-based

superalloys[18], $T$ = 0.5 Gb$^2$ is the dislocation line tension, $R$ is the real diameter of the particle, $h$ is the half-thickness of the precipitates, $\beta$ = 1/3 is a constant, and $f$ is the volume fraction of the precipitates. Using results from the HAADF–STEM analysis and dark-field TEM images (Supplementary Fig. 4b), the volume fraction $f$ of $\gamma''$ was calculated to be ~0.08. ε is the tetragonal lattice misfit, which was measured as 0.013, also from the HAADF-STEM images (Fig. 2b). With these data, the values of $\Delta\sigma_{pA}$ and $\Delta\sigma_{pC}$ were calculated from Eqs. 6 and 7 as 530 and 148 MPa, respectively; their sum is very close to the experimental value of $\Delta\sigma_{ps}$ (600 MPa). All these theoretical estimates are very consistent with our experimental results. We conclude that the strengthening by δ-lamellae and $\gamma''$ particles is over 1 GPa, which indicates that the independently controllable different precipitations strengthening mechanism plays a prominent role in enhancing the strength of this tungsten alloy.

We calculated the strengthening contributions from the stepwise coherent δ-lamella and $\gamma''$ particles to be approximately 425 and 678 MPa, respectively. Based on these estimates, Eq. 4 would suggest a total yield strength of ~2.1 GPa, which is in close agreement with the experimentally measured value of 2 GPa.

## Supplementary References


**1.** K. Lin, C. Hsu, S. Lin, Precipitation mechanism of an Mo Ni type intermetallic phase in W–27.0at%Mo–35.6at%Ni–17.6at%Fe, Int. J. Refract. Met. Hard Mater. **21**, 125–133 (2003). doi: 10.1016/S0263-4368(03)00008-8

**2.** Y. Yang, T. Chen, L. Tan, J. D. Poplawsky, K. An, Y. Wang, G. D. Samolyuk, K. Littrell, A. R. Lupini, A. Borisevich, E. P. George, Bifunctional nanoprecipitates strengthen and ductilize a medium-entropy alloy, Nature **595**, (2021). doi: 10.1038/s41586-021-03607-y

**3.** R. Feng, Y. Rao, C. Liu, X. Xie, D. Yu, Y. Chen, M. Ghazisaeidi, T. Ungar, H. Wang, K. An, P. K. Liaw, Enhancing fatigue life by ductile-transformable multicomponent B2 precipitates in a high-entropy alloy, Nat. Comm. **12**, 1-10 (2021). doi:10.1038/s41467-021-23689-6

**4.** Z.J. Zhang, M. M. Mao, J. Wang, H. Tian, B. Gludovatz, Z. Zhang, S. X. Mao, E. P. George, Q. Yu and R.O. Ritchie, Nanoscale origins of the damage tolerance of the high-entropy alloy CrMnFeCoNi, Nat. Comm. **6,** 10143 (2015). doi.org/10.1038/ncomms10143

**5.** E. Orowan, Symposium on *Internal Stress in Metals and Alloys*, Institute of Metals, London, 451–453 (1948).

**6.** M. Oblak, D.S. Duvall, D.F. Paulonis, An estimate of the strengthening arising from coherent, tetragonally-distorted particles, Mater. Sci. Eng. **13**, 51-56 (1974). doi: 10.1016/0025-5416(74)90020-2

**7.** J.Y. He, C.H. Zenk, X.Y. Zhou, S. Neumeier, D. Raabe, B. Gault, S.K. Makineni, On the atomic solute diffusional mechanisms during compressive creep deformation of a Co-Al-W-Ta single crystal superalloy, Sci. Rep. **8**, 3276 (2018). doi: 10.1016/j.actamat.2019.11.035

**8.** M. Sundararaman, P. Mukhopadhyay, S. Banerjee, Precipitation of the δ-Ni3Nb phase in two nickel base superalloys, Metall. Trans. A. **19**, 453-465 (1988). doi: 10.1007/BF02649259

**9.** D. Caillard, A. Couret, The Hall-Petch law investigated by means of in situ straining experiments in lamellar TiAl and deformed Al. Microsc. Res. Tech. **72**, 261–269 (2009) doi: 10.1002/jemt.20679

**10.** K. Ming, X. Bi, J. Wang, Realizing strength-ductility combination of coarse-grained $Al_{0.2}Co_{1.5}CrFeNi_{1.5}Ti_{0.3}$ alloy via nano-sized, coherent precipitates. Int. J. Plast. **100**, 177–191 (2018). doi: 10.1016/j.ijplas.2017.10.005

**11.** A. J. Ardell, Precipitation hardening. Metall. Trans. A **16**, 2131–2165 (1985). doi: 10.1007/BF02670416

**12.** R.W. Kozar, A. Suzuki, W.W. Milligan, J.J. Schirra, M.F. Savage, T.M. Pollock, Strengthening mechanisms in polycrystalline multimodal nickel-base superalloys. Metall. Mater. Trans. **A 40**, 1588–1603 (2009). doi: 10.1007/s11661-009-9858-5

**13.** W.F. Hosford, *Mechanical Behavior of Materials*, 1$^{st}$ ed., Cambridge University Press, New York, America, 2005.

**14.** L.M. Brown, R.K. Ham, Dislocation-particle interactions, in: A. Kelly, R.B. Nicholson

(Eds.), *Strengthening Methods in Crystals*, Elsevier, Amsterdam, 1971, 9-135. doi: 10.11470/oubutsu1932.41.652

**15.** M.X. Yang, F.P. Yuan, Q.G. Xie, Y.D. Wang, E. Ma, X.L. Wu. Strain hardening in Fe–16Mn–10Al–0.86C–5Ni high specific strength steel. Acta Mater. **109**, 213–222 (2016). doi: 10.1016/j.actamat.2016.02.044

**16.** H. Chen, X. Zi, Y. Han, J. Dong, S. Liu, C. Chen, Microstructure and mechanical properties of additive manufactured W-Ni-Fe-Co composite produced by selective laser melting. Int. J. Refract. Met. Hard Mater. **86**, 105–111(2020). doi: 10.1016/j.ijrmhm.2019.105111

**17.** P. Shi, W. Ren, T. Zheng, Z. Ren, X. Hou, J. Peng, P. Hu, Y. Gao, Y. Zhong, P.K. Liaw, Enhanced strength–ductility synergy in ultrafinegrained eutectic high-entropy alloys by inheriting microstructural lamellae, Nat. Comm. **10,** 498 (2019). doi: 10.1038/s41467-019-08460-2

**18.** F. He, D. Chen, B. Han, Q. Wu, Z. Wang, S. Wei, D. Wei, J. Wang, C.T. Liu, J. J. Kai. Design of D022 superlattice with superior strengthening effect in high entropy alloys. Acta Mater. **167,** 275-286 (2019). doi: 10.1016/j.actamat.2019.01.048

**19.** D. Caillard, A. Couret, The Hall-Petch law investigated by means of in situ straining experiments in lamellar TiAl and deformed Al. Microsc. Res. Tech. **72**, 261–269 (2009) doi: 10.1002/jemt.20679

**20.** O.N. Senkov, G.B. Wilks, J.M. Scott, D.B. Miracle. Mechanical properties of $Nb_{25}Mo_{25}Ta_{25}W_{25}$ and $V_{20}Nb_{20}Mo_{20}Ta_{20}W_{20}$ refractory high entropy alloys, Intermetallics **19,** 698-706 (2011). doi: 10.1016/j.intermet.2011.01.004

**21.** Inconel Alloy 718, http://www.specialmetals.com/documents/Inconelalloy718. pdf. Haynes 230 Alloy, http://www.haynesintl.com/pdf/h3060.pdf.
